# Metagenomic Analysis of Sediment Bacterial Diversity and Composition in Natural Lakes and Artificial Waterpoints of Tabuk Region in King Salman Bin Abdulaziz Royal Natural Reserve, Saudi Arabia

**DOI:** 10.3390/life14111411

**Published:** 2024-11-01

**Authors:** Yahya S. Al-Awthan, Rashid Mir, Basmah M. Alharbi, Abdulaziz S. Alatawi, Fahad M. Almutairi, Tamer Khafaga, Wael M. Shohdi, Amal M. Fakhry, Mashari M. Alatawi

**Affiliations:** 1 Department of Biology, Faculty of Science, University of Tabuk, Tabuk 71491, Saudi Arabia; b.alharbi@ut.edu.sa (B.M.A.); abalatawi@ut.edu.sa (A.S.A.); mm_alatawi@ut.edu.sa (M.M.A.); 2 Biodiversity Genomics Unit, Faculty of Science, University of Tabuk, Tabuk 71491, Saudi Arabia; 3 Department of Medical Laboratory Technology, Prince Fahad Bin Sultan Chair for Biomedical Research, Faculty of Applied Medical Sciences, University of Tabuk, Tabuk 71491, Saudi Arabia; rashid@ut.edu.sa; 4 Department of Biochemistry, Faculty of Science, University of Tabuk, Tabuk 71491, Saudi Arabia; falrabae@ut.edu.sa; 5 King Salman Bin Abdulaziz Royal Natural Reserve Development Authority, Riyadh 12213, Saudi Arabia; t.khafaga@ksrnr.gov.sa (T.K.); w.elsheikh@ksrnr.gov.sa (W.M.S.); 6 Botany and Microbiology Department, Faculty of Science, Alexandria University, Alexandria 21568, Egypt; amal.fakhry@alexu.edu.eg

**Keywords:** metagenome analysis, bacterial community, sediment, lakes, natural reserve, biodiversity

## Abstract

The Tabuk region is located in the northern part of Saudi Arabia, and it has an area of 117,000 km^2^ between longitudes 26° N and 29° N and latitudes 34° E and 38° E. King Salman Bin Abdulaziz Royal Natural Reserve (KSRNR) is the largest natural reserve in Saudi Arabia and covers about 130,700 km^2^. It represents a new tourist attraction area in the Tabuk region. Human activities around the lake may lead to changes in water quality, with subsequent changes in microenvironment components, including microbial diversity. The current study was designed to assess possible changes in bacterial communities of the water sediment at some natural lakes and artificial waterpoints of KSRNR. Water samples were collected from ten different locations within KSRNR: W1, W2, W3 (at the border of the royal reserve); W4, W5, W6, W7 (at the middle); and W8, W9, and W10 (artificial waterpoints). The total DNA of the samples was extracted and subjected to 16S rRNA sequencing and metagenomic analysis; also, the environmental parameters (temperature and humidity) were recorded for all locations. Metagenomic sequencing yielded a total of 24,696 operational taxonomic units (OTUs), which were subsequently annotated to 193 phyla, 215 classes, 445 orders, 947 families, and 3960 genera. At the phylum level, *Pseudomonadota* dominated the microbial communities across all samples. At the class level, *Gammaproteobacteria*, *Clostridia*, *Alphaproteobacteria*, *Bacilli*, and *Betaproteobacteria* were the most prevalent. The dominant families included *Enterobacteriaceae*, *Pseudomonadaceae*, *Clostridiaceae*, *Comamonadaceae*, and *Moraxellaceae*. At the genus level, *Pseudomonas*, *Clostridium*, *Acinetobacter*, *Paenibacillus*, and *Acidovorax* exhibited the highest relative abundances. The most abundant species were *Hungatella xylanolytica*, *Pseudescherichia vulneris*, *Pseudorhizobium tarimense*, *Paenibacillus* sp. *Yn15*, and *Enterobacter* sp. *Sa187*. The observed species richness revealed substantial heterogeneity across samples using species richness estimators, Chao1 and ACE, indicating particularly high diversity in samples W3, W5, and W6. Current study results help in recognizing the structure of bacterial communities at the Tubaiq area in relation to their surroundings for planning for environmental protection and future restoration of affected ecosystems. The findings highlight the dominance of various bacterial phyla, classes, families, and genera, with remarkable species richness in some areas. These results underscore the influence of human activities on microbial diversity, as well as the significance of monitoring and conserving the reserve’s natural ecosystems.

## 1. Introduction

The Tabuk region is located in the northern part of the Kingdom of Saudi Arabia, and it has an area of 117,000 km^2^ between longitudes 26° N and 29° N and latitudes 34° E and 38° E. King Salman bin Abdulaziz Royal Natural Reserve (KSRNR) is the largest natural reserve in the Kingdom of Saudi Arabia and covers about 130,000 km^2^. It is located in the north beside the Jordan–Saudi Arabia border. KSRNR is known for its pure nature, fresh air, geographic and heritage diversity, and rare monuments dating back to about 8000 BC. It consists of three primary conservation areas, which are Khunfah, Al-Tubayq, and Harrat Al-Harra [1,2]. The Tubaiq area is under the management of the King Salman bin Abdulaziz Royal Reserve Development Authority. The reserve is located in the northwest of the Kingdom, on the border with the Kingdom of Jordan. The total area of the reserve is approximately 12,200 square kilometers. The reserve is characterized by its rugged nature due to the presence of mountains on its western and central sides known as the Tabaiq Mountains, whose height reaches about 1388 m, in addition to reefs and valleys. There are also sandy and limestone sedimentary rocks, in addition to sandy areas on the eastern side of the reserve [2,3,4]. The vegetation cover in the reserve is somewhat weak as a result of overgrazing and logging. Acacia trees and some herbs and shrubs abound in the valleys. The most important animals found in the reserve are the ibex, the Arabian wolf, foxes, and wild rabbits. There are also some types of reptiles and endemic and migratory birds [2,3,4].

The microbial communities that are connected with freshwater sources in Royal Nature Reserves serve as the foundation for the food web and biogeochemical cycle of its ecosystem. Bodies of water represent natural boundaries between humans and other living organisms. They may be exposed to pollution from various sources of organic and inorganic pollutants, resulting in changes in water quality and threatening the surrounding ecosystem [5]. Furthermore, as water quality plays a major role in determining human health, pollutants in water, especially microorganisms, may pose a serious risk to human health, either directly or indirectly, through other environmental factors. Microorganisms play important roles in freshwater ecosystems through breaking down organic matter into essential nutrients and provide an important link in the food chain of freshwater systems [6]. A freshwater system’s water quality can be significantly altered by the variety of microorganisms found in lakes [7]). Therefore, the different types of lakes provide one of the most important sources of water for human and other organisms’ use. According to Han et al. [8], bacteria are the most significant member of the microbial community in aquatic ecosystems and play a vital role in several essential global ecological processes. The kind and concentration of bacteria can reveal details about the source of pollution and the quality of the water. Moreover, the establishment of bacterial communities in water bodies may help define how human activity affects water ecologies.

Traditional approaches to assessing microbial diversity in surface waters and sediments, such as cultivation-based techniques, have severe limitations. According to studies, typical culture methods can only collect less than 2% of the total microbial population in aquatic environments [9]. This constraint occurs because many microorganisms have specialized growth requirements that cannot be addressed under typical laboratory conditions. As a result, the great bulk of microbial diversity goes unexplored. Recent advances, such as metagenomic sequencing and diffusion-based integrative cultivation techniques, have revealed significantly greater microbial diversity and permitted the isolation of hitherto unknown taxa [10,11]. These contemporary techniques provide a more thorough understanding of microbial communities and their ecological significance, emphasizing the importance of moving beyond old methodologies in order to fully grasp microbial diversity in natural habitats.

The development of short-term and long-term water monitoring systems across various water bodies within the royal reserve is required to monitor the presence of some bacterial pathogens which indicate a low water quality. So, the biodiversity research could be used to gain a better understanding of the structure and function of the microbial community because it provides more information on the current bacteria [12,13,14,15]. Over the past two decades, microbial ecologists have used numerous techniques to study sediment microbial communities with microbial diversity coverage ranging from 5% for freshwater to 25% for sediment, but these techniques are effort- and time-consuming. In order to comprehend the composition and variety of microbial communities within a given environment, microbiologists have resorted to using rapid 16S ribosomal ribonucleic acid (rRNA) sequence-based techniques. The 16S rRNA gene has been effectively examined in sediments from all over the world. Moreover, Dahllof [16] notes that these methodologies are limited in their ability to offer comprehensive data on the structure and diversity index of microbial communities.

Metagenomic studies could be conducted to gain a deeper comprehension of the structure and function of the microbial community, as they offer a more detailed view of the current bacterial population. With the use of direct DNA extraction, metagenomics allows one to precisely identify the microbial structure and monitor the natural composition of microbiota [17]. A recent study found that bacterial diversity in biofilms on microplastics is greatly influenced by the lake’s trophic state. The presence of some bacteria, such as *Pseudomonas putida* and *Lacibacterium aquatile*, might indicate the type of contamination and overall water quality. Furthermore, the study discovered that microplastics can serve as vectors for infections such as *Escherichia coli*, regardless of the lake’s trophic state [18]. This information could shed light on how changes in water quality and pollution levels impact microbial communities. Bacterial diversity in water sediments can be significant due to the presence of both aquatic and terrestrial bacteria, including different soil species [19,20]. Over the past ten years, a number of studies have been carried out worldwide to examine the microbial community composition and the microbial biodiversity in sediments and water of lakes and rivers utilizing a metagenomics methodology [21,22,23,24,25,26,27,28,29,30,31,32]. In the current study, water samples were collected from both natural lakes and artificial waterpoints in KSRNR. Our initial goal was to look at the metagenomics of the bacterial communities in these water sediments to discover the richness and distribution of bacterial species across different areas within the reserve using extensive 16S rRNA sequencing and metagenomic analysis. This study sheds light on the different types of bacteria that live in these habitats, as well as their spatial distribution and how it connects to human activity in the area. Our findings add to a better understanding of KSRNR’s microbial ecology and highlight the possible effects of human activities on microbial diversity and ecosystem health. We believe that this study can provide new insights into the types of bacteria, their distribution in different areas within the royal reserve, and the relationship of distribution to human activities.

## 2. Methodology

### 2.1. Study Site and Sample Collection

**Figure 1:**
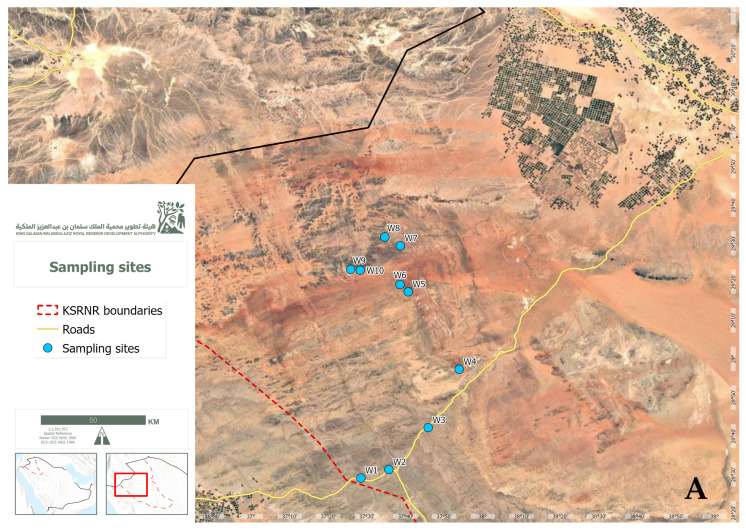

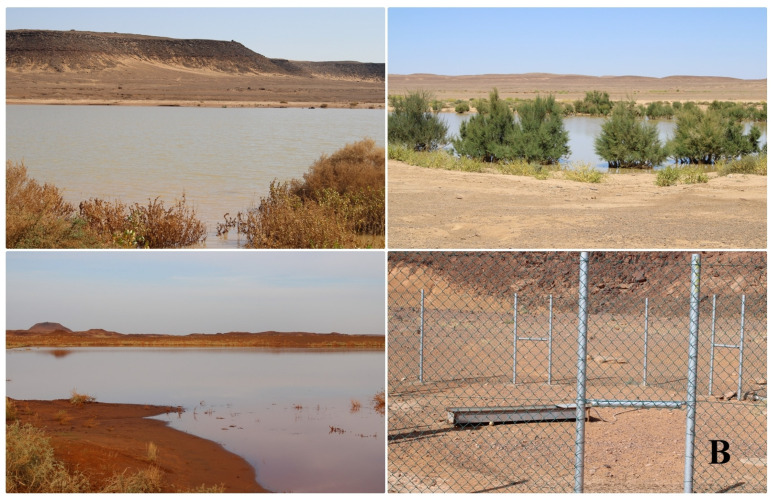
Map of the location of study area at the Tubaiq area of KSRNR (**A**). The sampling sites of the study at the Tubaiq area of KSRNR, Sshowing three Nnatural lakes and one Aartificial Waterpoint (**B**).

The water samples were collected from 10 locations in King Salman Bin Abdulaziz Royal Reserve, Tabuk, Saudi Arabia in Nov 2023: W1 (28°29′06.5″ N 37°28′25.9″ E), W2 (28°31′16.7″ N 37°35′41.2″ E), and W3 (28°42′12.3″ N 37°45′52.9″ E) were at the border of the royal reserve and subjected to human activities; few plants were observed. W4 (28°57′18.4″ N 37°53′53.0″ E), W5 (29°17′24.7″ N 37°40′42.0″ E), W6 (29°19′17.0″ N 37°38′35.3″ E), and W7 (29°29′23.4″ N 37°38′38.8″ E) were at the middle of the royal reserve and subjected to little human activities; small grasses were noticed. W8 (29°31′37.3″ N 37°34′40.4″ E), W9 (29°23′12.3″ N 37°25′47.9″ E), and W10 (29°23′01.9″ N 37°28′16.6″ E) were artificial waterpoints far away from human activities without plants around the area. A map of the locations in the study area is shown in Figure 1. From each location, three replicate samples (1 L each) of water were collected. The samples were stored on ice during transportation to our lab at the Biodiversity Genomics Unit, Faculty of Science, University of Tabuk, Saudi Arabia. The samples were kept at −80 °C before DNA extraction and quantification, and then to the Unipath Lab, Ahmedabad, India for DNA sequencing and metagenomics. The temperature and humidity were recorded for each site using a digital thermometer and a hygrometer.

### 2.2. Isolation and Quantitative Analysis of DNA

Genomic DNA was extracted from the collected samples using the Alexgen Soil DNA Extraction Kit (CAT No-AG-SD50), followed by quantification using a Qubit^®^ 4.0 fluorometer (Waltham, MA, USA). To amplify the 16S rRNA gene, the universal primers 8F and 1492R were employed, and the resulting amplicons were visualized on a 1.8% agarose gel electrophoresis.

### 2.3. Library Preparation

Paired-end sequencing libraries were constructed using the Twist Bioscience DNA Library Kit (cat104119, Gateway Blvd, South San Francisco, CA, USA) for Illumina^®^ with a 50 ng DNA input. Enzymatic shearing fragmented DNA into smaller segments, followed by end repair and A-tailing to prepare fragments for adapter ligation. Illumina-specific adapters were ligated to both ends of the DNA fragments to facilitate library binding, PCR amplification, and sequencing primer binding. To optimize yield, a high-fidelity PCR amplification step was performed using HiFi PCR Master Mix. The quality and quantity of the amplified libraries were assessed using an Agilent TapeStation 4150 system with High Sensitivity D1000 ScreenTape^®^ according to the manufacturer’s protocol.

### 2.4. Cluster Generation and Sequencing

Following Qubit concentration determination and TapeStation profile analysis, the library was loaded onto an Illumina NovaSeq 6000 system for cluster generation and sequencing. Paired-end sequencing was employed to sequence template fragments bidirectionally. Library molecules hybridized to complementary adapter oligos on the paired-end flow cell. The adapter design facilitated selective cleavage of forward strands post reverse strand re-synthesis, allowing for sequencing from the opposite end of the fragment.

### 2.5. Data Generation

Raw sequence data generated by the NovaSeq6000 platform underwent demultiplexing to separate individual samples. Before de novo assembly, adapter sequences and low-quality reads (QV < 20) were removed from the dataset through quality filtering. The cleaned reads were subsequently assembled using MEGAHIT v1.2.9 [5,33], a specialized metagenome assembler designed for handling large and complex metagenomic datasets.

### 2.6. Gene Prediction

Gene prediction was performed on the assembled scaffolds using Prodigal (v2.6.3) [6,34] with the metagenome gene prediction mode. The predicted gene sequences were then utilized for subsequent taxonomic and functional analyses.

### 2.7. Taxonomic Annotation

Taxonomic classification of metagenomic reads was performed using Kaiju, a rapid and sensitive metagenome classifier [7] This tool leverages the Burrows–Wheeler transform algorithm to identify maximal exact matches at the protein level within a reference database comprising annotated protein-coding genes from a collection of microbial genomes. The default reference database employed in this study consisted of complete genomes sourced from NCBI RefSeq, supplemented by the microbial subset of the NCBI non-redundant protein database (nr), with the optional inclusion of fungi and microbial eukaryotes.

Kaiju operates by translating metagenomic reads into all six potential reading frames and subsequently searching for maximal exact matches (MEMs) of amino acid sequences against the specified protein database [28,32]. The algorithm, based on a modified backward search within the Burrows–Wheeler transform, efficiently identifies these matches. For reads exhibiting matches to multiple database sequences, Kaiju assigns the taxonomic identifier corresponding to the lowest common ancestor (LCA) within the taxonomic hierarchy [28,35]. In this study, sequences generated by Prodigal were subjected to Kaiju analysis [28,34]. The standalone version of Kaiju was utilized with the following parameters: database—nr; sequence low complexity filter—enabled; run mode—greedy; minimum match length—11 amino acids; minimum match score—75; and allowed mismatches—5.

### 2.8. Diversity Analysis

Alpha-diversity indices are an essential tool for describing and comparing biodiversity. Alpha diversity, a measure of species richness and evenness within a sample, was calculated based on operational taxonomic unit (OTU) abundance data derived from Kaiju classification results [32]. The OTU table served as the input for alpha diversity estimation, which was conducted using the *estimate_richness* function within the R package phyloseq (version 1.48.0) [8,36,37]. This analysis yielded estimates for several diversity indices: Chao1, ACE, Shannon, Simpson, Inverse Simpson, and Fisher.

### 2.9. Statistical Analysis and Data Visualization

All statistical analyses were performed using R, version 4.3.2 [16]. Data manipulation and visualization were executed with the aid of the tidyverse package (version 2.0.0) [12] ggplot2 package (version 3.4.4) [13]. corrplot package (version 0.92) [14], and complexheatmap package (version 2.18.0) [15,33,34,36,38] within the R environment [37,39].

## 3. Results

### 3.1. Study Site Characteristics

The ten sediment samples analyzed in this study were sourced from two distinct lacustrine environments: natural and artificial. Geographic coordinates revealed a clear spatial separation between the two lake types. Natural lakes exhibited median longitude and latitude values of 28.8° and 37.7°, respectively, with ranges of 28.5° to 29.3° for longitude and 37.5° to 37.9° for latitude. In contrast, artificial waterpoints displayed more concentrated geographic distributions, with median longitude and latitude values of 29.4° and 37.4°, respectively, and narrower ranges of 29.4° to 29.5° and 37.4° to 37.6°.

Elevation profiles also differentiated the two lake types. Natural lakes demonstrated a median elevation of 814 m, ranging from 756.5 to 924.2 m. Artificial waterpoints, on the other hand, were situated at higher elevations, with a median of 869 m and a range of 782.5 to 920.2 m.

Climatic parameters exhibited notable differences between natural lakes and artificial waterpoints. Natural lakes displayed a median humidity of 27.5%, ranging from 19% to 32%, while artificial waterpoints had a lower median humidity of 19.5%, ranging from 18% to 26%. Temperature variations were also observed, with natural lakes experiencing a median temperature of 29.5 °C (range: 27 °C to 37 °C) and artificial waterpoints exhibiting a higher median temperature of 32.5 °C (range: 32 °C to 34 °C). Table 1 provides an overview of the characteristics of the study site.

### 3.2. Taxonomic Composition of Sediment Microbial Communities

**Figure 2:**
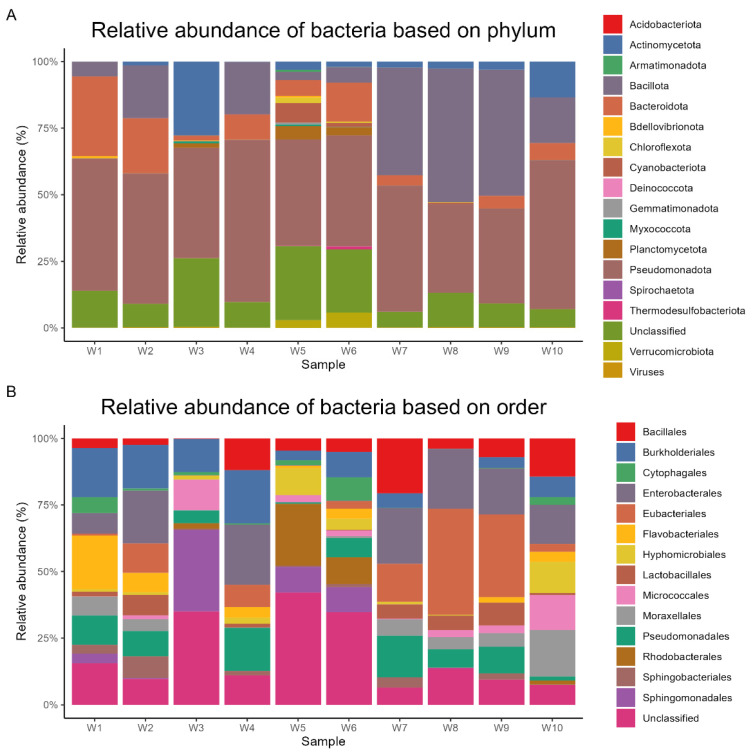
Relative abundance of dominant bacterial phyla and orders in lake sediment samples.

**Figure 3:**
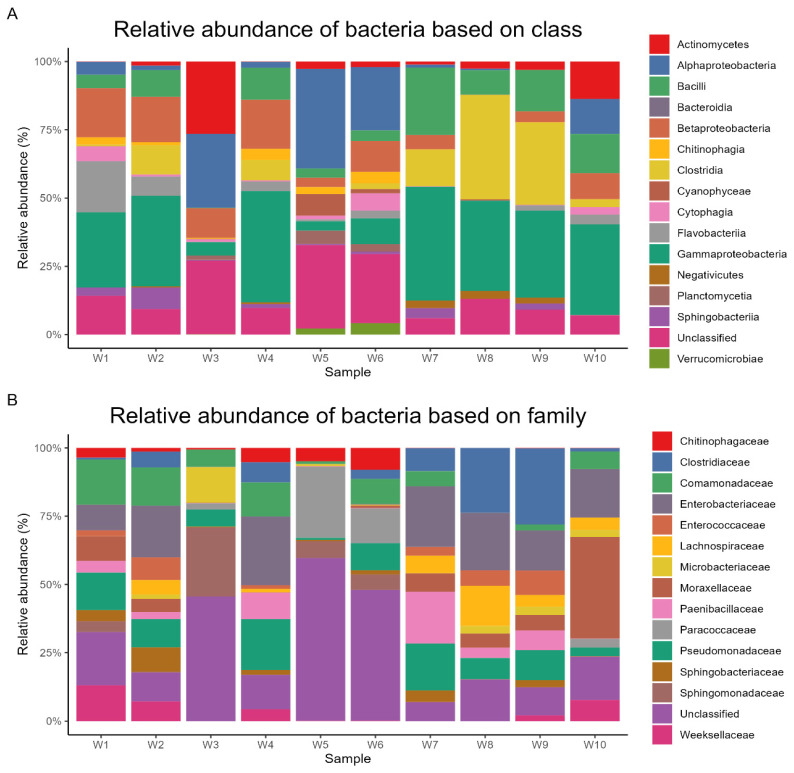
Relative abundance of dominant bacterial classes and families in lake sediment samples.

**Figure 4:**
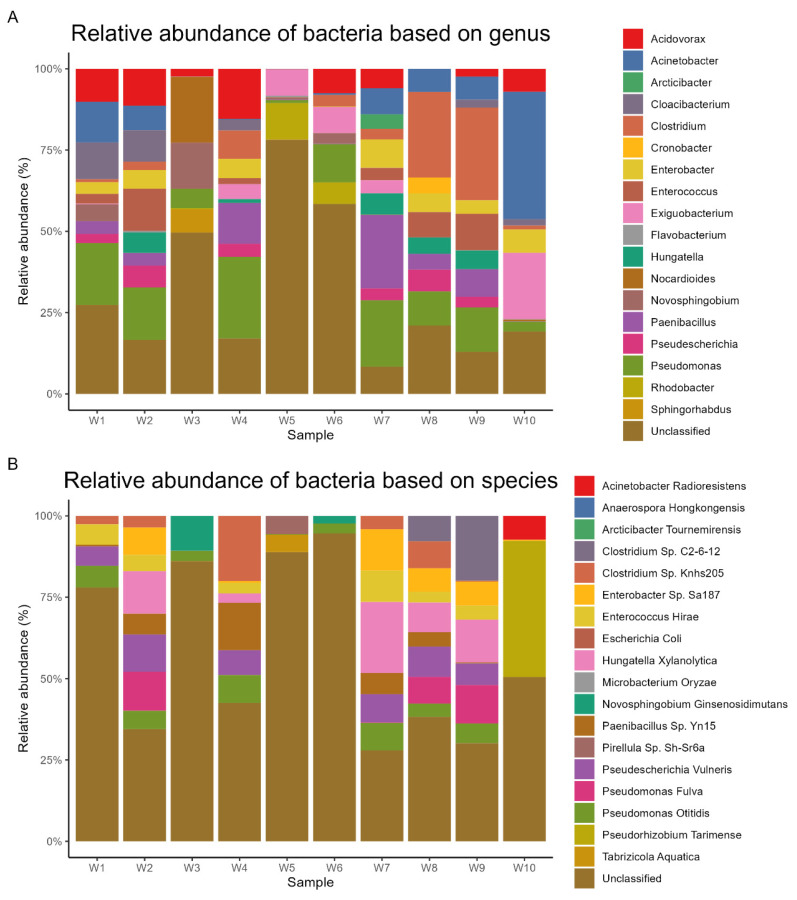
Relative abundance of dominant bacterial genera and species in lake sediment samples.

Metagenomic sequencing yielded a total of 24,696 operational taxonomic units (OTUs), which were subsequently annotated to 193 phyla, 215 classes, 445 orders, 947 families, and 3960 genera. At the phylum level, *Pseudomonadota* dominated the microbial communities across all samples, representing an average relative abundance of 44% (range: 32.7–58.8%). *Bacillota* (20%, 0.2–49.3%), *Bacteroidota* (9%, 0.1–29.2%), Actinomycetota (5%, 0.1–27%), and *Planctomycetota* (1%, 0–4.9%) were the other predominant phyla. The most abundant orders were *Enterobacterales* (11%, 0.1–21%), *Eubacteriales* (10%, 0–37%), *Burkholderiales* (8%, 0–16.9%), *Pseudomonadales* (7%, 0.4–14.1%), and *Bacillales* (6%, 0.1–18.5%). At the class level, *Gammaproteobacteria* (24%, 3.1–39.6%), *Clostridia* (10%, 0.1–37.1%), *Alphaproteobacteria* (10%, 0.1–32.4%), *Bacilli* (9%, 0.1–23.4%), and *Betaproteobacteria* (9%, 0–17.1%) were the most prevalent. The dominant families included *Enterobacteriaceae* (9%, 0–18.6%), *Pseudomonadaceae* (7%, 0.3–14%), *Clostridiaceae* (6%, 0–24%), *Comamonadaceae* (5%, 0–11.5%), and *Moraxellaceae* (4%, 0–15.7%). At the genus level, *Pseudomonas* (7%, 0.3–14%), *Clostridium* (5%, 0–19.5%), *Acinetobacter* (4%, 0–13.6%), *Paenibacillus* (4%, 0–15.5%), and *Acidovorax* (3%, 0–8.4%) exhibited the highest relative abundances. Finally, the most abundant species were *Hungatella xylanolytica* (2%, 0–4.5%), *Pseudescherichia vulneris* (1%, 0–3.1%), *Pseudorhizobium tarimense* (1%, 0–5.5%), *Paenibacillus* sp. *Yn15* (1%, 0.1–3.2%), and *Enterobacter* sp. *Sa187* (1%, 0–2.6%). The relative abundances of dominant bacterial taxa at different taxonomic levels are visually represented based on phylum (Figure 2A), order (Figure 2B), class (Figure 3A), family (Figure 3B), genus (Figure 4A), and species (Figure 4B).

### 3.3. Dominant Microbial Genera per Sample

To elucidate the specific microbial composition within each sediment sample, a detailed analysis of the most abundant genera was conducted. Sample W1 was characterized by the predominance of *Pseudomonas* (9%), followed by *Flavobacterium* (7%) and *Acinetobacter* (6%). In sample W2, *Pseudomonas* again emerged as the dominant genus (8%), with *Enterococcus* (7%) and *Acidovorax* (6%) as the subsequent most abundant taxa. A distinct microbial profile was observed in sample W3, with *Nocardioides* as the dominant genus (10%), followed by *Novosphingobium* (7%) and *Yonghaparkia* (6%). In contrast, sample W4 exhibited a dominance of *Pseudomonas* (14%), succeeded by *Acidovorax* (8%) and *Paenibacillus* (7%). A shift in microbial community structure was evident in samples W5 and W6. While *Leptolyngbya* was the most abundant genus in sample W5 (5%), followed by *Rhodobacter* (4%) and *Exiguobacterium* (3%), sample W6 showed a lower microbial diversity, with *Pseudomonas* as the dominant genus (5%), accompanied by *Exiguobacterium* (3%) and *Acidovorax* (3%). A return to Gram-positive dominance was observed in samples W7 and W8. *Paenibacillus* was the most prevalent genus in sample W7 (15%), followed by *Pseudomonas* (14%) and *Enterobacter* (6%). Similarly, *Clostridium* dominated sample W8 (16%), with *Pseudomonas* (6%) and *Enterococcus* (5%) as secondary constituents. This trend continued in sample W9, where *Clostridium* remained the most abundant genus (20%), followed by *Pseudomonas* (9%) and *Enterococcus* (8%). Finally, sample W10 showed a predominance of *Acinetobacter* (14%), with *Exiguobacterium* (7%) and *Pseudorhizobium* (6%) as the subsequent dominant genera. These findings underscore the heterogeneity of microbial communities across the sampled sediments and highlight the potential influence of various environmental factors on bacterial composition. In Table 2, the distribution of the top three genera per sample is illustrated.

### 3.4. Sample Correlation and Clustering

**Figure 5:**
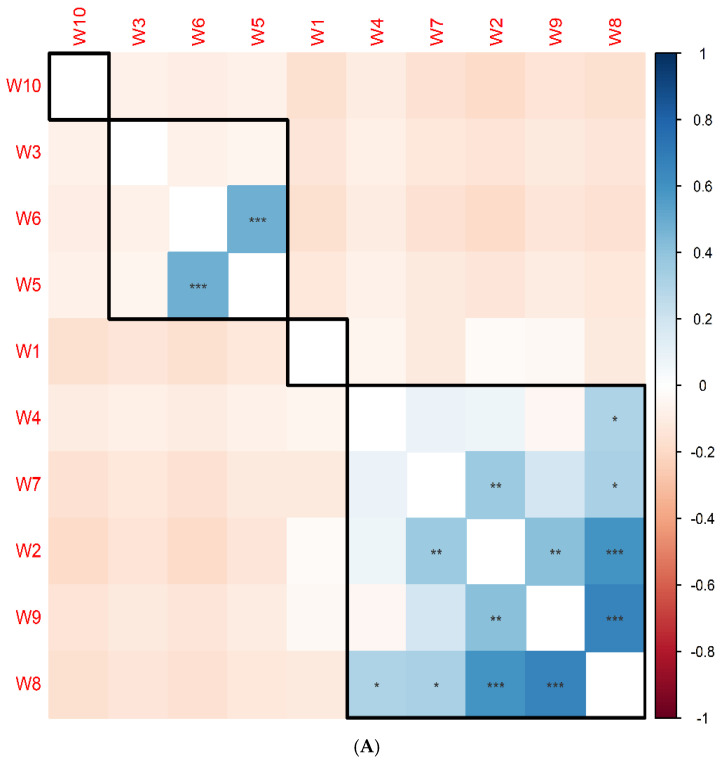

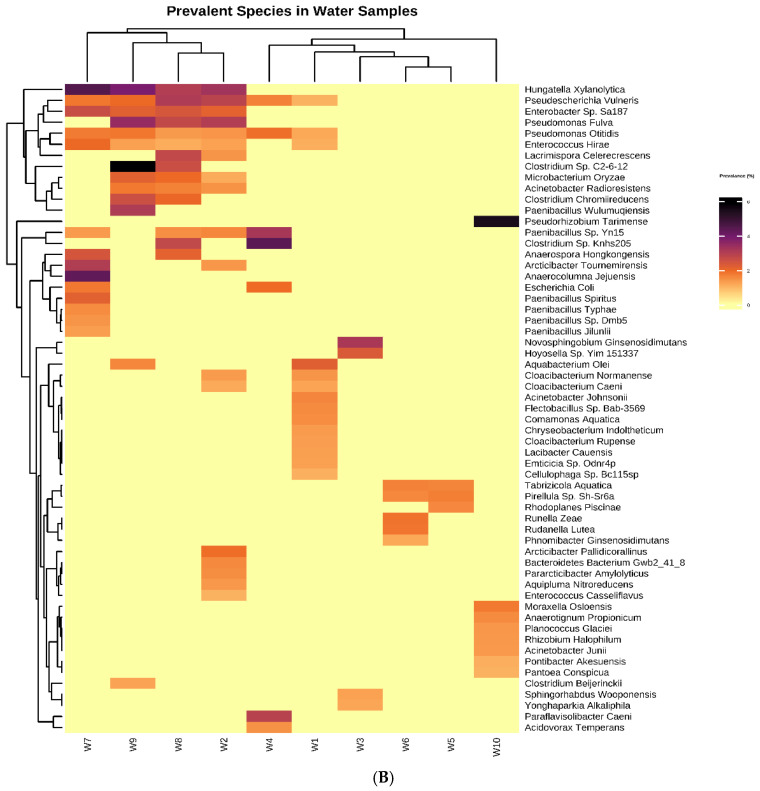
(**A**) Correlation matrix illustrating the relationships between sediment samples based on bacterial species prevalence. (Pearson’s correlation. * *p* < 0.05, ** *p* < 0.01, *** *p* < 0.001) (**B**) Hierarchical clustering and heatmap visualizing the clustering of samples and the distribution of top prevalent species.

To investigate the relationships among sediment samples based on their bacterial community composition, correlation and clustering analyses were performed using bacterial species prevalence data. Pairwise correlation analysis using Pearson’s correlation method revealed significant positive correlations between specific samples. Notably, samples W8 and W9 exhibited the strongest correlation (rho = 0.7, *p* = 8 × 10^−9^), suggesting a high degree of similarity in their bacterial community structure. Additionally, sample W8 displayed a substantial correlation with sample W2 (rho = 0.6, *p* = 7 × 10^−7^). Furthermore, samples W5 and W6 were found to be significantly correlated (rho = 0.5, *p* = 8 × 10^−5^). Hierarchical clustering analysis grouped the samples into distinct clusters based on their bacterial community profiles. Samples W2, W4, W7, W8, and W9 formed a cohesive cluster, indicating a relatively close relationship among their microbial communities. In contrast, samples W3, W6, and W5 clustered together, suggesting a shared bacterial community pattern. Due to their unique microbial profiles, samples W1 and W10 did not cluster with any other samples. The correlation matrix and heatmap with dendrogram visually represent the relationships between samples and the clustering patterns, respectively (Figure 5A,B).

### 3.5. Clusters of Orthologous Groups (COG) Functional Annotation

**Figure 6:**
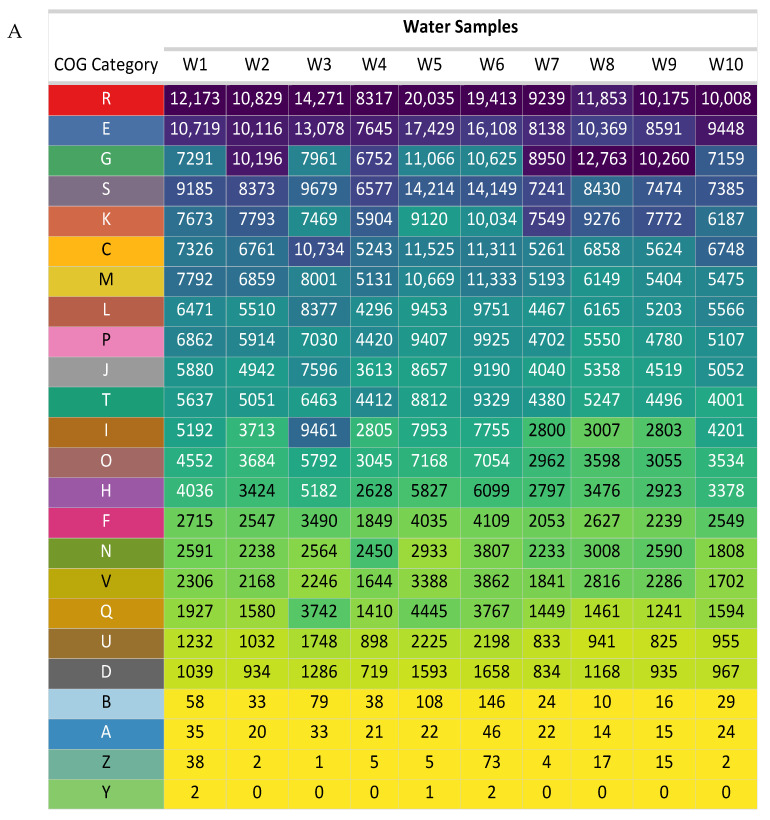

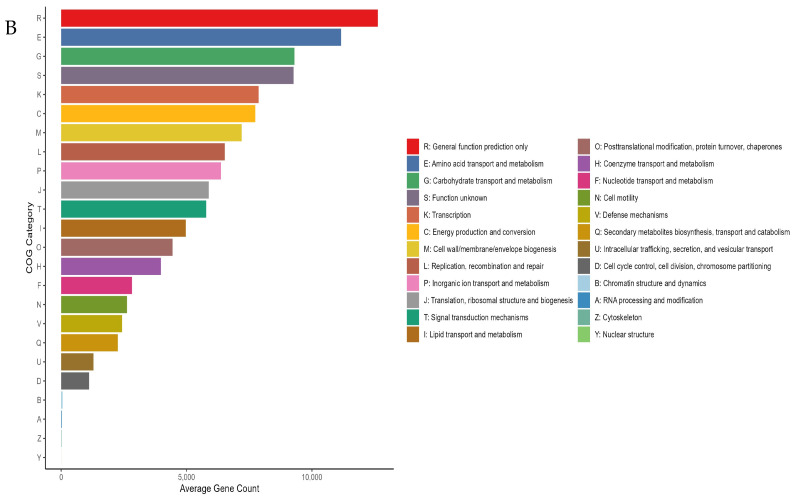
(**A**) Enriched OTUs categorized by COG functional groups. (**B**) Average count of OTUs associated with different COG categories.

To elucidate the functional profiles of the identified protein clusters, COG functional annotation was performed. Based on the average number of clustered proteins assigned to each COG category across all samples, a hierarchical classification of COG functions was established. The top ten COG categories, in descending order of average protein abundance, were as follows: R: General function prediction only (average proteins = 12,631), E: Amino acid metabolism and transport (average proteins = 11,164), G: Carbohydrate metabolism and transport (average proteins = 9302), S: Function unknown (average proteins = 9271), K: Transcription (average proteins = 7878), C: Energy production and conversion (average proteins = 7739), M: Cell wall/membrane/envelope biogenesis (average proteins = 7201), L: Replication and repair (average proteins = 6526), P: Inorganic ion transport and metabolism (average proteins = 6370), and J: Translation (average proteins = 5885). The distribution of COG categories across individual samples and the enrichment of specific COG categories are visually represented in Figure 6A,B, respectively.

The comparative analysis of COG category distribution across individual water samples offers valuable insights into the functional diversity and adaptive strategies of microbial communities in natural and artificial environments. Consistent with their fundamental roles in core cellular processes, categories associated with general functional prediction (R) and amino acid metabolism and transport (E) were consistently abundant across most samples. However, significant variations in COG category profiles were observed, indicative of functional adaptations to specific ecological niches. Notably, the artificial waterpoints (W8–W10) exhibited a higher prevalence of carbohydrate metabolism and transport (G) categories, while natural lakes (W1–W7) demonstrated a dominance of lipid metabolism (I). Additionally, artificial waterpoints displayed greater abundances of transcription (K) and cell motility (N) COG categories, suggesting potential adaptations to anthropogenic influences or varying environmental conditions. In contrast, natural lakes were characterized by higher levels of cell wall biogenesis (M), inorganic ion transport and metabolism (P), and post-translational modification, protein turnover, and chaperone functions (O), suggesting a greater emphasis on cellular structure and maintenance. At the sample-specific level, W3, W5, and W6 exhibited elevated abundances of R, E, and C (energy production and conversion) categories, indicating active growth and metabolic processes. Conversely, lipid metabolism (I), post-translational modification (O), coenzyme metabolism (H), and nucleotide metabolism (F) were enriched in W3, W5, and W6 but depleted in W4, W7, W8, and W9, suggesting unique functional adaptations or environmental pressures within these specific samples.

These findings provide insights into the predominant functional attributes of the microbial communities inhabiting the sediment samples. The enrichment of specific COG categories in certain samples may indicate adaptations to specific environmental conditions or ecological niches.

### 3.6. Microbial Alpha Diversity

To characterize the microbial diversity within each sediment sample, a suite of alpha diversity indices was employed. Observed species richness, a direct count of OTUs, revealed substantial heterogeneity across samples, ranging from 3069 to 12,217 OTUs. Species richness estimators, Chao1 and ACE, corroborated these findings, indicating particularly high diversity in samples W3, W5, and W6. To account for both species richness and evenness, the Shannon and Simpson indices were calculated. These metrics generally indicated moderate to high diversity across samples, with sample W5 demonstrating the most pronounced diversity. Fisher’s alpha index, another measure of species’ richness, aligned with the aforementioned indices in identifying samples W3, W5, and W6 as harboring the most diverse microbial communities.

In contrast, sample W7 consistently exhibited the lowest values across all alpha diversity indices, suggesting a comparatively less diverse microbial community. Collectively, these results underscore the significant variation in microbial diversity among the sediment samples, highlighting the influence of environmental factors on microbial community structure. Table 3 provides a comprehensive summary of the alpha diversity observed in the water samples under study, presenting the values of all diversity indices utilized for the analysis.

## 4. Discussion

KSRNR is the largest protected area in the Middle East, and consists of three primary conservation areas, i.e., Khunfah, Tubaiq, and Harrat Al-Harra [1,2]. Tubaiq is located within the Tabuk region in the north of Saudi Arabia. Freshwater areas typically feature microbial taxa that differ from those found in terrestrial and marine areas [35]. Microbial communities play a crucial role in maintaining the health of aquatic ecosystems because of their close relationship to biogeochemical processes like the transportation of materials and energy, the breakdown of organic matter, and the recycling of nutrients [40,41]. A significant degree of diversity in these communities improves their stability and functionality [42]. However, other ecological parameters and the microenvironment of the microbial communities have a significant impact on their abundance [43]. The primary impact of biodiversity loss on ecosystem functioning is the threat it poses to several ecosystem services that are essential to human well-being. In the current study, the locations were selected from a map of lakes provided by the Royal Reserve Authority, based on differences in the surrounding landscape and preference in being utilized by wildlife that could have an impact on their microenvironment. The water samples from natural lakes and artificial waterpoints were collected, and the differences among the microbial communities in the ten sites were studied.

The current study showed that the elevation profiles were differentiated within the two lake types. In addition, climatic parameters exhibited notable differences between natural and artificial waterpoints. Natural lakes exhibited higher median humidity and lower median temperatures, while artificial waterpoints exhibited lower median humidity and higher median temperatures. These results were consistent with a previous report [44]. The salient distinctions between artificial and natural waterbody features underscore the significance of the waterbody filter with respect to export to the sediment ecosystem. Water chemistry can be changed by variations in clarity, which can impact lake ecosystem diversity and productivity [45].

Metagenomic analysis of sediment bacterial diversity indicated variations in the composition at taxonomic levels of bacterial communities across the ten different locations in lakes of Tabuk and that the surrounding conditions could have a relation impact. Metagenomic data were subsequently annotated to 193 phyla, 215 classes, 445 orders, 947 families, and 3960 genera. At the phylum level, *Pseudomonadota* dominated the microbial communities across all samples, representing an average relative abundance of 44%, followed by *Bacillota* (20%), *Bacteroidota* (9%), Actinomycetota (5%), and *Planctomycetota* (1%). Similar studies were in line with our results [32,46,47,48]

In cold climates [49,50] and freshwater habitats [51], *Pseudomonadota* and *Bacteroidota* frequently have a dominant position. These bacterial phyla were found in numerous investigations of lake ecosystems [52,53,54]. In addition, Shen et al. [47] reported that *Pseudomonadota* was the dominant bacterial phylum in most of the sediment samples of collapsed lakes in Huaibei, China. A further study investigated the bacterial communities found in the silt of 13 freshwater lakes on the Yunnan Plateau. Zhang et al. [21] discovered that Proteobacteria and Bacteroidetes constituted a significant portion of the bacterial communities observed in the sediment. Nevertheless, the major classes and their proportions differed significantly between lakes, influenced by the nitrogen content of the sediment.

To properly connect the structure and function of communities revealed by metagenomic research, it is imperative to investigate whether the phylogenic composition of metagenomic libraries matches the original microbial composition. The presence of several microbial families and classes in the water sediment samples used in this investigation supported by similar previous studies within other regions inside the Kingdom of Saudi Arabia [25,30,55].

The most common orders in our study area were *Enterobacterales* (11%), *Eubacteriales* (10%), *Burkholderiales* (*Betaproteobacteria*) (8%), *Pseudomonadales* (7%), and *Bacillales* (6%). In freshwater environments, bacteria are essential to the breakdown and cycling of nutrients. Research has demonstrated that *Burkholderiales* and *Bacteroidota* are prevalent in freshwater settings and play a role in the decomposition of organic materials [6,53]. The existence and activity of *Bacteroidota* emphasize their importance in preserving the balance of ecosystems as well as their possible effects on the health of people and other animals in contaminated aquatic systems.

The most common microorganisms at the class level were *Gammaproteobacteria* (24%), *Betaproteobacteria* (9%), *Bacilli* (9%), and *Clostridia* (10%). Previous published articles demonstrated the dominance of proteobacteria in Taif River water [56], in water and sediments of the Apies River, South Africa [24], and the dominance of *Bacilli* in hot spring sediments of Saudi Arabia [57,58]. Another study showed the dominance of proteobacteria in Pangong lake, India [26]. Another recent study showed that *Gammaproteobacteria*, *Betaproteobacteria*, *Alphaproteobacteria*, and Actinomycetia were the dominant class in the Beas River, Kangra, India [31]. Freshwater forms are more likely to contain *Betaproteobacteria*, *Alphaproteobacteria* Bacteroidetes, Actinobacteria, and Verrucomicrobia [59]

In the current metagenomic analysis, *Enterobacteriaceae* (9%), *Pseudomonadaceae* (7%), *Clostridiaceae* (6%), *Comamonadaceae* (5%), and *Moraxellaceae* (4%) were the leading families. Similar results were documented in different published articles [31,32,60,61,62]. Birds transfer microbes to water bodies based on environmental pollutants and eating habits, which may vary throughout the year [60]. The presence of *Enterobacteriaceae* and *Pseudomonadaceae* in most of the studied locations may be due to contamination of lake water with animal feces where the hot conditions in these areas force animals to get into water to reduce their body temperature. However, further studies are needed to focus on host–microbiome interactions in aquatic ecosystems.

At the genus level, *Pseudomonas* (7%), *Clostridium* (5%), *Acinetobacter* (4%), *Paenibacillus* (4%), and *Acidovorax* (3%) had the highest relative abundance. The majority of these bacterial species are common aerobic residents of soil, sediment, and freshwater, and they are well known for their ability to breakdown chemical contaminants. The *Pseudomonas* were found in most of the current sediment samples at higher percentages, while *Clostridium* was found only in artificial waterpoints (Table 2). There are many studies within the Kingdom of Saudi Arabia that concluded with similar results. For example, Li et al. [56] reported the dominance of *Pseudomonas* in water samples and sediments of the Taif River. Also, Al-Quwaie [63] reported the highest occurrence of *Pseudomonas* in the rhizospheres of some desert plants. Similar results were documented at the Kokemäenjoki River, Finland [64].

Finally, the most abundant species were *Hungatella xylanolytica* (2%), *Pseudescherichia vulneris* (1%), *Pseudorhizobium tarimense* (1%), *Paenibacillus* sp. *Yn15* (1%), and *Enterobacter* sp. *Sa187* 1%, (0–2.6%). Previous published articles demonstrated the dominance of *Paenibacillus* sp. in hot spring sediments [57], desert halophytes [65], and different soil samples [66] of Saudi Arabia. In addition, a recent study carried by Miguel et al. [67] showed that *Pseudescherichia vulneris* was among the most common species found under metagenomic investigation in the soil samples.

Pairwise correlation analysis indicated substantial positive correlations among specific samples. For example, samples W8 and W9 had the greatest correlation, indicating a significant degree of similarity in bacterial community structure. Also, sample W8 displayed a substantial correlation with sample W2. In addition, samples W5 and W6 were found to be significantly correlated. The reason for this connection may be due to the similarity of the natural terrain and climatic conditions of those sites, in addition to the type of water in those ponds, whether natural or artificial. Azli et al. [68] indicates that many bacterial taxa are highly correlated within the samples of the study area. For a clear representation, see (Figure 5A,B), where the correlation matrix and heatmap with dendrogram visually illustrate the associations between samples and clustering patterns, respectively.

COG functional annotation was used to better understand the functional characteristics of the discovered protein clusters. The average number of clustered proteins assigned to each COG category across all samples was used to create a hierarchical classification of COG functions. Figure 6A,B show the distribution of COG categories across various samples, as well as the enrichment of specific COG categories. These findings shed light on the primary functional characteristics of the microbial communities that occupy the sediment samples. The enrichment of various COG categories in certain samples could suggest adaptations to specific environmental circumstances or ecological niches [69,70,71].

A set of alpha diversity indices was used to characterize the microbial diversity in each water sediment sample. The observed species richness revealed significant variation between samples, with numbers ranging from 3069 to 12,217 OTUs. These findings were verified by species richness estimators Chao1 and ACE, which showed particularly high diversity in samples W3, W5, and W6. Many of metagenomics investigations found strong positive linear associations between bacterial species richness and functional gene richness [72,73]. In these investigations, species richness was calculated using 16S rRNA gene amplicon sequencing, whereas functional richness was estimated using Metagenomics Rapid Annotations.

According to Xia et al. [32], the Yellow River in China has a generally higher level of bacterial diversity than other rivers. This suggests that the high concentration of suspended particulate sediment plays a significant role in regulating bacterial diversity and community structure in aquatic environments. Similarly, Louca et al. [74] employed a taxon-assigned function approach to demonstrate a positive link between the number of functional groupings found in oceanic microorganisms and species diversity. Baeshen et al. [75] recently studied the soil microbiome diversity associated with some halophytic plants within Jeddah Seacoast, Saudi Arabia. They analyzed the bacterial diversity and richness and discovered that the samples differed greatly depending on the number of OTUs. The results showed that the bacterial abundance and diversity increased significantly along with the succession of halophyte vegetation [33,34,36,38,39,76]. The linkages between soil bacterial populations and the succession of halophyte plants were better understood as a result of the findings, which can aid in the development of effective plans to reduce CO_2_ emissions and improve carbon sequestration [37,39] The findings demonstrated that the succession of halophyte vegetation was substantially accompanied by an increase in bacterial diversity and abundance [77,78,79]. When determining the taxonomic profiles of whole biological communities, we highly advise relying solely on metagenomic techniques. Therefore, in order to develop an environmental management system and plan for the future restoration of affected ecosystems, our findings will aid in understanding the factors influencing the water microbiome.

## 5. Conclusions

The microbial communities that relate to freshwater sources in Royal Nature Reserves serve as the foundation for the food web and biogeochemical cycle. The current study was designed to assess possible changes in bacterial communities of the water sediment at some natural and artificial waterpoints of KSRNR. In the present investigation, 16S rRNA metagenomics were used to identify the sediment bacterial diversity and composition in 10 different Lakes of King Salman Bin Abdulaziz Royal Reserve, Tabuk, Saudi Arabia in order to establish a clear database of the bacterial communities and the biodiversity of the water system and try to correlate these data with human activities near the lakes. Our results indicated that differences in the microbial community structure at the studied locations are most likely consequences of the human activity. Therefore, our findings will help in understanding factors influencing the water microbiome at the natural lakes and artificial waterpoints of King Salman Bin Abdulaziz Royal Reserve for the development of an environmental management system and planning for the future restoration of affected ecosystems.

## Figures and Tables

**Table 1:** Physiochemical characteristics of the study sites and sediment samples.

Samples	Type	Latitude (°)	Longitude (°)	Elevation (M)	Humidity (%)	Temperature (°C)
W1	Natural lake	28.48515	37.47386	924.2 m	30	27
W2	Natural lake	28.52132	37.59478	882.4 m	32	27
W3	Natural lake	28.70341	37.7647	838.2 m	26	33
W4	Natural lake	28.9551	37.89805	788.8 m	19	37
W5	Natural lake	29.2902	37.67833	756.5 m	29	29
W6	Natural lake	29.32138	37.64315	763.5 m	25	30
W7	Natural lake	29.48984	37.64411	782.5 m	26	32
W8	Artificial Waterpoint	29.52703	37.5779	865.4 m	20	32
W9	Artificial Waterpoint	29.38676	37.42997	920.2 m	18	34
W10	Artificial Waterpoint	29.38387	37.47128	872.4 m	19	33

**Table 2:** Distribution of the top three bacterial genera in each sediment sample.

	Water Samples									
Genus	W1	W2	W3	W4	W5	W6	W7	W8	W9	W10
*Acinetobacter*	**6%**	0%	0%	0%	0%	0%	0%	0%	0%	**14%**
*Exiguobacterium*	0%	0%	0%	0%	**3%**	**3%**	0%	0%	0%	**7%**
*Pseudorhizobium*	0%	0%	0%	0%	0%	0%	0%	0%	0%	**6%**
*Pseudomonas*	**9%**	**8%**	0%	**14%**	0%	**5%**	**14%**	**6%**	**9%**	0%
*Flavobacterium*	**7%**	0%	0%	0%	0%	0%	0%	0%	0%	0%
*Enterococcus*	0%	**7%**	0%	0%	0%	0%	0%	**5%**	**8%**	0%
*Acidovorax*	0%	**6%**	0%	**8%**	0%	**3%**	0%	0%	0%	0%
*Nocardioides*	0%	0%	**10%**	0%	0%	0%	0%	0%	0%	0%
*Novosphingobium*	0%	0%	**7%**	0%	0%	0%	0%	0%	0%	0%
*Yonghaparkia*	0%	0%	**6%**	0%	0%	0%	0%	0%	0%	0%
*Paenibacillus*	0%	0%	0%	**7%**	0%	0%	**15%**	0%	0%	0%
*Leptolyngbya*	0%	0%	0%	0%	**5%**	0%	0%	0%	0%	0%
*Rhodobacter*	0%	0%	0%	0%	**4%**	0%	0%	0%	0%	0%
*Enterobacter*	0%	0%	0%	0%	0%	0%	**6%**	0%	0%	0%
*Clostridium*	0%	0%	0%	0%	0%	0%	0%	**16%**	**20%**	0%

Genus ≥ 1% are highlighted in bold.

**Table 3:** Alpha diversity indices for each sediment sample.

Sample ID	Observed	Chao1	ACE	Shannon	Simpson	Fisher
W1	5491	9964.96	10,248.02	5.47	0.99	1259.15
W2	5122	8922.56	9344.67	5.05	0.98	1170.13
W3	10,119	16,569.74	17,163.93	6.50	0.99	2552.76
W4	4201	7953.63	8255.81	5.31	0.97	1042.07
W5	12,217	18,841.19	19,157.80	6.80	0.99	2996.31
W6	11,603	18,809.67	19,798.13	6.42	0.99	2759.19
W7	3069	6528.03	7006.79	4.46	0.97	654.51
W8	4257	8332.38	8186.91	4.93	0.98	903.91
W9	4082	8280.35	8688.01	4.72	0.97	889.43
W10	4766	9354.21	9740.23	5.53	0.98	1150.61

## Data Availability

The data presented in this study are available on request from the corresponding author.
